# Effects of Dietary Babassu Oil or Buriti Oil on Nutrient Intake and Total Tract Digestibility, and Abomasal Digesta Fatty Acid Profile of Lambs

**DOI:** 10.3390/ani12091176

**Published:** 2022-05-04

**Authors:** Nítalo Machado, Michelle Parente, Rui Bessa, Henrique Parente, Ruan Gomes, Ricardo Pinho, Daniele Ferreira, Anderson Zanine, Juliany Costa, Susana Alves

**Affiliations:** 1 Center of Environment and Agriculture Science, Federal University of Maranhão, Chapadinha 65500-000, Brazil; michellemrn14@gmail.com (M.P.); hnparente@hotmail.com (H.P.); ruanmourao@gmail.com (R.G.); dany_dosanjos@yahoo.com.br (D.F.); anderson.zanine@ufma.br (A.Z.); julianymendes40@gmail.com (J.C.); 2 CIISA, AL4Animals, Faculty of Veterinary Medicine, University of Lisboa, 1300-477 Lisboa, Portugal; rjbbessa@fmv.ulisboa.pt (R.B.); susanaalves@fmv.ulisboa.pt (S.A.); 3 Federal Institution of Education, Science and Technology of Ceará, Crato 63115-500, Brazil; ricardo-zootec@hotmail.com

**Keywords:** lipid stress, medium-chain fatty acids, ruminal biohydrogenation

## Abstract

**Simple Summary:**

This research tested the effects of adding babassu oil (450 g/kg C12:0 of total fatty acids—FA) or buriti oil (750 g/kg C18:f total FA) to the diet of lambs on intake, nutrient digestibility, FA profile of abomasal digesta content and biohydrogenation patterns in digestive content. Both are widely available in the Northeast of Brazil and Amazon region. Our results provide evidence that the babassu supplemented diet promotes greater stress to the ruminal bacteria (due to the high concentration of C12:0), changing the normal biohydrogenation of polyunsaturated FA (PUFA) in the rumen, and the FA concentration that flows to the abomasum, compared to the buriti oil supplemented diet, which provided similar results to the non-supplemented diet.

**Abstract:**

Our current understanding of the effect of medium-chain FA (MCFA) rich vegetable oils on ruminant nutrition is limited. We assessed the effects of babassu or buriti oil addition to the diet of lambs on intake, nutrient digestibility, FA profile of abomasal digesta content and biohydrogenation (BH) patterns in digestion. The experimental diets were defined by the addition of babassu oil or buriti oil to the diet, as follows: (1) non-supplemented diet (CON); (2) 40 g/kg of babassu oil (BAO, rich in C12:0); and (3) 40 g/kg of buriti oil (BUO, rich in *c*9 18:1), on a dry matter (DM) basis. During the last five days of the feedlot, samples of orts and feces were individually collected to determine the nutrient and FA digestibility. At the end of the experiment, animals were slaughtered, and the abomasal digesta was collected, freeze-dried and used for FA determinations conducted by gas chromatography. The BAO diet decreased the DM (*p* = 0.014) and nutrient intake. The lambs fed BUO had the greatest FA intake, followed by the BAO and CON diets. However, BAO increased total FA digestibility, compared with CON, but did not differ from BUO. The BAO diet extensively changed the FA composition of abomasal digesta when compared with both the CON and BUO diets. The BAO diet also increased C12:0 and C14:0, the sum of PUFA and the BH intermediates FA, including the t-10-18:1 but decreased the C18:0 in abomasal digesta. The BUO addition had the greatest total-FA and C18:0 and the lowest biohydrogenation intermediate content in abomasal digesta. The BH was less complete with the BAO diet and a large increase in *t*10-18:1 and of *t*10-/*t*11-18:1 ratio was observed, which indicates the occurrence of *t*10 possibly shifted rumen BH pathways, probably as a response to bacterial membrane stress induced by the greater C12:0 concentration in the rumen.

## 1. Introduction

Lipid supplementation increases the energy density of diets and thus can increase ruminant’s energy intake and production [1]. However, feeding ruminants with unprotected lipids might lead to depression of fiber digestibility and ultimately dry matter (DM) intake due to the toxic effects of fatty acids (FA), particularly of unsaturated FA, on rumen microbiota [2].

The unsaturated FA are extensively isomerized and hydrogenated by rumen microbiota, in a metabolic process known as ruminal biohydrogenation (BH) [3]. The BH is responsible for the saturation in ruminant tissues and milk lipids, as well as for the presence of BH intermediates, mostly trans FA [4]. In general, the BH rate (i.e., the disappearance of dietary PUFA) is increased with polyunsaturated FA (PUFA) [5,6], but the BH completeness is decreased and BH intermediates accumulate [6]. 

Medium-chain FA (MCFA) are known to have antibacterial effects [7,8] and may also exert an inhibitory effect on ruminal BH, as mentioned in previous studies [8,9]. Although MCFA are saturated FA and thus not a substrate for the BH, an increase in trans FA in the meat of sheep fed vegetable oil, with a greater amount of C12:0, has been reported [10], even without a significant quantity of precursors. This concept allows us to suggest the hypothesis that the bacteria allow the adaptation of membrane fluidity in stressful conditions, caused by the high concentration of C12:0, as an accumulation of membrane-toxic compounds [11,12].

Babassu (*Attalea speciosa* Mart ex) oil, derived from babassu coconut, has a high concentration of MCFA, especially C12:0 (i.e., 45% of total FA, [10]). Thus, its FA composition is less prone to oxidative degradation than other vegetable oils with higher PUFA, increasing the storage time in farm conditions. On the other hand, buriti (*Mauritia flexuosa* L.) oil, a product of buriti palm grinding and fruit extraction, contains large proportions (75%) of oleic acid. Both of these are widely available in the Northeast of Brazil and the Amazon region, and because of that, the interest in using these oils as a fat source in animal diets by local farmers has increased. 

We hypothesize that the babassu supplemented diet, rich in MCFA, will promote greater stress to ruminal bacteria (due to the high concentration of C12:0), disturbing the normal biohydrogenation of PUFA in the rumen, and consequently the FA concentration that flows to the abomasum. Considering that MCFA are not precursors of trans C18 FA, they are ideal for testing the hypothesis that lipid stress per se induces the formation of trans C18 FA. To test these hypotheses, we assessed the effects of adding babassu oil or buriti oil to the diet of lambs on intake, nutrient digestibility, FA profile of abomasal digesta content and biohydrogenation patterns in digestive content.

## 2. Materials and Methods

### 2.1. Location and Animal Management

The experiment was conducted at the Small Ruminant Sector, Center of Agrarian and Environmental Sciences, Federal University of Maranhão, located in Chapadinha, MA (03°44′33″ S, 43°21′21″ W), Brazil. Animal handling followed the guidelines recommended by the Animal Care and Use Committee of the same institution (Process number 23115009965/2014–80).

A total of 21 Dorper × Santa Inês growing wethers, with initial mean body weight (BW) of 18 ± 3 kg and a mean age of 123 ± 12 days old were used, as previously reported [10]. After day 50 of the experimental period, the lambs remained in the pens for five days for the collection of digestibility data.

### 2.2. Experimental Design and Diets

The experimental design consisted of a randomized complete block, with three treatments and seven blocks, which were defined according to the weight and age of the lambs at the beginning of the experiment. The animals were individually housed in covered pens with a concrete floor, and experimental diets were randomly allocated to pens in each block; thus, the animal was the experimental unit. 

The treatment consisted of addition of babassu (Florestas Brasileiras S.A., Itapecuru Mirim, MA, Brazil) and buriti oils (handmade by local producers) to the diet, maintaining the content of supplemental FA at 4% DM. The oils were added to a basal diet that contained 70% DM of concentrate and 30% DM of forage (Tifton hay). The treatments were as follows: (1) basal diet without added oil (CON), (2) 40 g/kg DM of babassu oil (BAO), and (3) 40 g/kg DM of buriti oil (BUO). The experimental diets were formulated according to National Research Council to meet the requirements of a growing lamb [1]. The chemical and FA composition of diets are shown in Table 1. The fatty acid profile of babassu and buriti oils is listed in Table 2.

During diet formulation, corn was coarsely ground using a grinder (Trapp, TRF 80, Jaraguá do Sul, SC, Brazil) and mixed with soybean meal, urea, limestone and mineral premix. The oil was added to the concentrate immediately before feeding. The concentrate and hay were separately weighed using an electronic scale (Welmy, BCW 6/15/30, Santa Bárbara d’Oeste, SP, Brazil), manually mixed and offered daily as a total mixed ration.

The amounts of feed offered and refused during the five days were recorded to adjust feed offered for 10% refusal (digestibility data). 

### 2.3. Sample Collection and Chemical Analysis

To collect the total feces, appropriate bags were attached to the animals, and samples were collected twice a day, at 07:30 and 16:00 h. They were then weighed, homogenized, and approximately 100 g/kg of the total sample volume was stored in a freezer at −20 °C for subsequent laboratory analysis.

During the five days of feces collection, the samples of experimental diets and orts were also collected and were thawed and pooled by lamb. All samples collected were dried at 55 °C for 72 h in a forced-air oven, and then were ground through a 1 mm Wiley Mill screen (Marconi, Piracicaba, SP, Brazil). Then, the DM (Method 934.01), ash (Method 942.05), ether extract (Method 954.05) and total nitrogen (N; Method 968.06) were determined according to the Association of Official Analytical Chemists (AOAC) [13]. Crude protein was calculated by multiplying the total nitrogen by 6.25. Neutral detergent fiber, assayed with a heat stable amylase and expressed exclusive of residual ash (aNDFom), [14]. Total carbohydrates (Total CHO) were obtained by the following equation: Total CHO (%) = 100 − (%CP + %EE + %Ash). Non-fiber carbohydrates (NF-CHO) were estimated according to the equation: NF-CHO = 100 − (%NDF + %CP + %EE + %Ash). The metabolizable energy (ME) of the diets were estimated using the Small Ruminant Nutrition System, v. 1.8.6 [15].

After 50 days of feedlot, animals were slaughtered, without fasting, in accordance with the norms established by the Regulation of Brazilian Industrial and Sanitary Inspection of Products of Animal Origin. Immediately after slaughter, abomasal digesta content was collected from each lamb and representative samples were frozen, and freeze-dried in the same week of slaughter, and then, vacuum packed (SINBO, DZ-280, Beijing, China) and stored at −20 °C until further FA determination. The pH in rumen fluid was measured on whole rumen contents strained through four layers of cheesecloth.

The fatty acid composition of the experimental ingredients of diets, orts, feces and abomasal digesta samples were analyzed as fatty acid methyl ester (FAME) derivatives. Thus, freeze-dried samples were directly transesterified by a reaction with sodium methoxide in methanol (0.5 M) at 50 °C for 10 min followed by a reaction with HCl in methanol (1.25 M) at 80 °C for 15 min [6]. Additionally, 1 mL of methyl nonadecanoate (1 mg.mL^−1^ in n-hexane) was added before transesterification, to be used as the internal standard. FAME were analyzed by GC with flame ionization detection (GC-FID) using a Shimadzu GC 2010-Plus (Shimadzu, Kyoto, Japan) equipped with an SP-2560 (100 m × 0.25 mm, 0.20 m film thickness, Supelco, Bellefonte, PA, USA) capillary column. The chromatographic conditions for GC-FID were as follows: injector and detector temperatures were maintained at 250 °C and 280 °C, respectively; the initial oven temperature of 50 °C was held for 1 min, increased at 50 °C/min to 150 °C and held for 20 min, increased at 1 °C/min to 190 °C, and then increased at 2 °C/min to 220 °C and held for 40 min. Helium was used as the carrier gas at a flow rate of 1 mL/min and 1 μL of the sample (1–2 mg FAME/mL) was injected, and the split ratio was 50:1. Identification of FAME was achieved by comparison of the retention times with those of authentic standards (37 Component FAME Mix from Supelco Inc., Belfont, PA, USA) and published chromatograms [16]. The identification of FAME were confirmed by gas chromatography–mass spectrometry (GC–MS) using a GC-MS QP 2010 Plus chromatograph (Shimadzu Corp., Kyoto, Japan). The GC-MS capillary column and the GC conditions were similar to the GC-FID analysis and the mass spectrometer conditions were as follows: ion source temperature, 200 °C; interface temperature, 240 °C; emission voltage, 70 V. 

### 2.4. Biohydrogenation Estimate and Digestibility Calculation

The biohydrogenation (BH) estimates (%) for c9-18:1, 18:2 n-6 and 18:3 n-3 were obtained using the diminishing abundance of these FA, proportional to the sum of C18-carbon FA, between the diet and abomasal digesta (1) [17], assuming that no losses of C18 FA occurred in the gastric compartments [18].

(1) $$\mathrm{BH}\;(\%)=\frac{\mathrm{FA}\;\mathrm{Diet}\;-\;\mathrm{FA}\;\mathrm{Abomasum}}{\mathrm{FA}\;\mathrm{Diet}}\;\times \;100$$

where, BH (%): is the estimate of the disappearance of FA between the diet and the abomasal digesta; FA Diet: is the FA in the diet as a percentage of total C18 FA; FA Abomasum: is the FA in abomasal digesta as percentage of total C18 FA.

The biohydrogenation completeness (%) was estimated considering the maximum 18:0 in abomasal digesta (2) [6], assuming a complete biohydrogenation of the C18 FA from the diet.

(2) $$\mathrm{BH}\;\mathrm{Completeness}\;(\%)=\frac{18:0\;\mathrm{Abomasum}}{\mathrm{Maximum}\;18:0\;\mathrm{Abomasum}}\;\times \;100$$

where, maximum 18:0 Abomasum = (c9-18:1 Diet − c9-18:1 Abomasum) + (18:2n-6 Diet − 18:2n-6 Abomasum) + (18:3n-3 Diet − 18:3n-3 Abomasum) + 18:0 Diet.

The total tract apparent digestibility of nutrients and FA was obtained by the proportional relationship between the ingesta and excreta amounts.

### 2.5. Statistical Analysis

The validity of the residual normality assumption was verified by the Shapiro-Wilk test. It was carried out using ANOVA by the MIXED procedure of the Statistical Analysis System [19] for a randomized complete block design, according to the model: Y_ij_ = µ + B_i_ + D_j_+ ε_ij_, where µ = mean, B_i_ = the random effect of the block, D_j_ = the fixed effect of the diet, and ε_ij_ = experimental error. Lamb was included as random effect in the statistical model. The homogeneity of variances were checked for each variable and, when needed, models accommodating heterogeneous variances were fitted, following the procedures described by Milliken and Johnson [20].

Significance was declared at *p* < 0.05. When significant treatment effects were found, a post hoc analysis using the Tukey procedure was applied to identify significant differences (*p* < 0.05) between the least square means. The mean variability of each treatment was evaluated and when necessary, the individual standard errors of the means were presented in the tables.

## 3. Results

### 3.1. Intake and Total Tract Digestibility

The BAO diet reduced (*p* = 0.014) DMI and consequently also reduced the CP (*p* = 0.024), NDF (*p* = 0.008), TC (*p* = 0.005), and NFC intake (*p* = 0.004) of the lambs in relation to other diets. However, the metabolizable energy intake (*p* = 0.280) and apparent digestibility (*p* < 0.05) of nutrients did not differ among the diets (Table 3). Ruminal pH was higher (*p* < 0.001) when lambs were fed BUO followed by the CON and BAO diet (Table 3).

The intake of total FA, the sum of C18:0, c9-18:1, and c11-18:1 were higher (*p* < 0.001) in BUO, followed by BAO and then CON (Table 4). As result of the absence or minor content of 12:0 in the CON and BUO diets, respectively, the 12:0 intake and digestibility were only calculated in BAO supplemented lambs. 

Lambs fed BAO had the greatest intake (*p* < 0.001) and digestibility (*p* = 0.005) of 18:0, while 16:0 intake was greater (*p* < 0.001) for BUO, followed by BAO and then CON. The oil addition in the diet did not change the 18:2 n-6 intake (*p* = 0.231) or the digestibility (*p* = 0.121). No or only minor amounts of 18:3 n-3 were recovered in feces resulting in high apparent digestibility, especially in BUO (*p* < 0.001; Table 4). 

Lambs fed with BUO had the greatest (*p* < 0.001) intake and digestibility of c9-18:1, the main FA in buriti oil, followed by the BAO and then CON diets. The sum of C18 FA (SC18 FA) digestibility did not differ among treatments (*p* = 0.725), however lambs fed BAO had higher (*p* = 0.047) total FA digestibility compared with CON but did not differ from BUO.

### 3.2. Abomasal Digesta Fatty Acid Profile and Ruminal Biohydrogenation

The abomasal content contained several FA that were not present in the diet as the t6/t7/t8-18:1, t9-18:1; t10-18:1, t11-18:1, t12-18:1, t15-18:1, c12-18:1, c13-18:1, t16/c14-18:1, c15-18:1, t11, c15-/t10, c15-18:2 and oxo-18:0 (Table 5).

**Figure 1:**
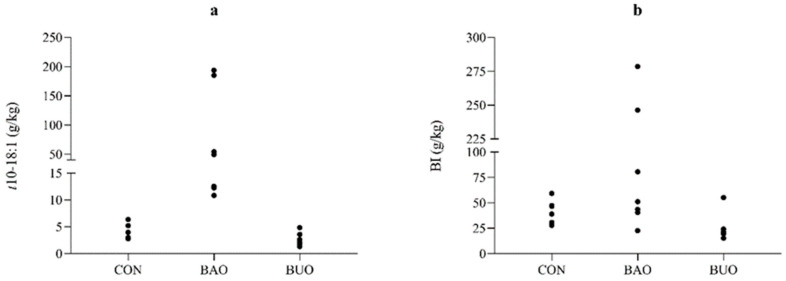
Proportion of t-10 18:1 (**a**) and total biohydrogenation intermediates (BI) (**b**) in abomasum content of sheep fed control diet (CON) or supplemented diets with babassu (BAO) or buriti (BUO) oil.

Although some of the trans-FA were reduced (*p* < 0.05) when lambs were supplemented with vegetable oils, the t10-18:1 (Figure 1a) increased in a greater proportion when lambs were fed BAO (*p* = 0.002) content, as well as increased trans MUFA (*p* = 0.003, Figure 1b) and t10-18:1/t11-18:1 ratio (*p* = 0.002) in relation to other diets. The t10-18:1 comprised about 66% of all biohydrogenation intermediates (BI) in BAO treatment and only ≈10% in the other treatments. The second most abundant BI was t11-18:1, which also differed among treatments (*p* = 0.027), being higher in the CON treatment (18.1 g/kg FA) than in the supplemented diets (mean of 6.1 g/kg FA) (Table 5).

The BAO supplementation increased the proportion of PUFA (*p* = 0.041) and decreased the proportion of 18:0 (*p* = 0.001) in the abomasal content of lambs compared with the CON and BUO diets (Table 5). The 16:0 (*p* = 0.041) and 18:2 n-6 (*p* = 0.028) were less and SFA (*p* = 0.001) content was greater in abomasum of lambs fed BUO compared with BAO. The abomasum content of lambs fed BUO had the least 18:3 n-3 (*p* = 0.047) proportion and BAO had the greatest oxo-18:0 (*p* = 0.002) followed by BUO and CON (Table 5).

Furthermore, the total BCFA also decreased (*p* = 0.028) with oil supplementation, regardless of the source added in relation to the CON diet. This is due to the reduction in minor BCFA (i-14:0, i-15:0, a-15:0, i-17:0 and a-17:0 + c9-16:1) in abomasal content of supplemented lambs. The lambs fed BUO increased (*p* = 0.003) the total FA abomasal content (Table 5).

The estimated C18 BH of UFA and BH completeness are presented in Table 6. The addition of vegetable oils increased (*p* < 0.024) the c9-18:1 and decreased the biohydrogenation of 18:2 n-6 (*p* = 0.001) and 18:3 n-3 (*p* < 0.001). The BAO diet had the lowest (*p* = 0.006) BH completeness.

## 4. Discussion

The BAO diet clearly depresses DMI when compared with the CON and BUO diets. In this study, there was no effect on ME intake, the differences in DMI probably result from a compensatory response [21]. According to the theory of intake regulation, ruminants control their intake of food to achieve an approximate constancy of metabolizable energy intake [22]. 

High-fat diets often reduce the growth of cellulolytic microorganisms leading to reduced digestibility of fiber and delayed rumen particle passage rate [1]. Fat sources with high contents of PUFA and MCFA, especially lauric acid (12:0) are known to harm the predominant rumen fiber-degrading bacterial population [23]. Despite this, the present study demonstrated that adding up to 40 g/kg DM of babassu oil did not depress the NDF digestibility, probably because the fat content was within acceptable limits of DM [1]. 

The BAO fed lambs presented the lowest rumen pH, which seems contradictory considering that they also had the lowest DM and NFC intake. Lauric acid, and coconut oil, have been proposed as powerful defaunating agents [24,25]. The rumen ciliates actively engulf starch granules and thus contribute to a more gradual starch fermentation and prevention of acidotic events, being positively correlated with rumen pH [26]. 

Although it was not evaluated in this study, it is possible that the protozoan population could be depressed in BAO fed lambs. In this situation, the starch would be quickly fermented by amylolytic bacteria, resulting in pH reduction, as observed in lambs fed BAO. 

Concerning the apparent digestibility of individual FA, sometimes negative values were found (Table 4). This generally occurred when FA were consumed in amounts below 0.1 g/day, as observed for 16:0 and c11-18:1 digestibility. For 18:0 digestibility, this occurred even when lambs were fed greater amounts (ranging from 0.67 to 1.14 g/100 g), which means the formation of this FA in the gastrointestinal tract, resulted from the net increase in this FA due to the extensive BH of unsaturated C18 FA.

The apparent digestibility of c9-18:1 in BUO fed lambs was also very high, however, most of the c9-18:1 is hydrogenated into 18:0 that is less digestible in the small intestine than shorter saturated FA as previously established [27,28,29]. The apparent digestibility of 12:0 was almost complete (994 g/kg) in BAO. Contrasting with the longer FA that are absorbed exclusively in the small intestine, the 12:0 is absorbed both in the rumen (circa 30%) and in the small intestine [30]. The 12:0 absorbed by the small intestine is mostly transferred as NEFA to portal vein blood [31], and then extensively oxidized by ß-oxidation in the liver, as reported in rats [32], whereas the longer FA are re-esterified and exported in chylomicrons into the lymph.

The great diversity of FA in abomasal content is mostly explained by the rumen lipid metabolism that involves extensive lipolysis followed by BH of unsaturated FA, de novo synthesis of microbial FA and dietary FA that escaped rumen metabolization. Most of the trans-18:1, except t10-18:1 in BAO lambs, are present in a lower proportion in the oil supplemented diets than in the CON.

The percentage of biohydrogenation of dietary C18 unsaturated FA using the FA composition of abomasum digesta, which has been appointed as representative of the overall process and easily interpretable, was estimated [6,17]. Despite this, it is frequently reported that increased C18 PUFA intake increases C18 PUFA biohydrogenation rates [5]; however, our abomasal estimates are not consistent with this.

The 18:2n-6 intake did differ among the diets and the 18:3n-3 intake was only slightly greater for BUO and only c9-18:1 showed relevant intake differences, ranging from ≈1 g/d with CON, 5 g/d with BAO and up to 31 g/d with BUO. Both 18:2n-6 and 18:3n-3 are the BH substrates that originate more diversity of trans intermediates. Although the generation of trans isomers from c9-18:1 has been established [33], pH conditions below 6.5 increase the conversion of oleic acid to stearic acid, probably because of a shift in the bacterial population [34]. The BH of c9-18:1 in the BUO fed lambs was very extensive and very complete, with the formation of 18:0. 

The large increase in t10-18:1 in the abomasal content of BAO is also surprising, particularly considering the low intake of PUFA with this diet and the reduced BH of 18:2n-6 and 18:3n-3. The large increase in t10-18:1 implies either the occurrence of a possible shift in the BH pathways or a reduction in the completeness of such pathways to allow for the accumulation of t10-18:1 instead of 18:0. Consequently, the total trans MUFA in abomasum content of sheep fed BAO also increased. 

As reviewed [3], in intensively produced ruminants, it is frequent that the main rumen BH pathway, that generates t11-18:1 as the major trans-octadecenoic intermediate, is replaced by a–usually minor–pathway that generates t10-18:1 as the major trans-octadecenoic; this is known as the t10-shift. The occurrence of the t10-shift is favored by diets that also favor low rumen pH as well as diets with higher concentrate:forage ratio, and low rumen effective fiber [35].

As discussed above, the results found in this research for rumen pH and a possible reduction in protozoa, might favor the occurrence of the t10-shift, because protozoa are a major predator of rumen bacteria. Despite the type of trans-octadecenoate generated, the low BH completeness observed in BAO lambs is also relevant, and thus the consequent accumulation of the trans-18:1 intermediates, resulted in a greater trans-18:1 in meat from BAO fed lambs [10]. 

The less complete BH is generally due to the inhibition of the last reductive step of the BH pathway, which for a long time was believed to be catalyzed only for few specialized (i.e., group B/*Butyrivibrio proteoclasticus* strains) biohydrogenating bacteria very sensitive to the toxic effects of PUFA [36]. Thus, the classic explanation for the more incomplete BH and trans-FA accumulations have been the effect of stress agents, often UFA, on these group B bacteria. 

Nevertheless, a clear association between trans-FA accumulation and *Butyrivibrio proteoclasticus* strains remains elusive [37] and it is now evident that most biohydrogenation activity must be conducted by uncultured bacteria [38]. An alternative explanation for the less complete BH is that increasing trans-18:1 in the rumen helps the rumen microbiota handle stress factors including high PUFA, low pH and tannins [39,40]. Due to its antibacterial effect, the presence of a greater 12:0 concentration in the rumen of BAO fed lambs will be a stress factor [8,9], leading to the accumulation of trans-18:1, even without an expressive dietary load of BH substrates. 

Incorporation of trans-18:1 in membranes is a widespread stress bacterial response and cis to trans isomerization has been reported as a mechanism enabling several non-rumen gram-negative bacteria to adapt to several forms of environmental stress and permit the adaptation of membrane fluidity to change chemical or physical parameters of the cellular environment [11,12]. In the rumen, the BH seems to fill the role of supplying trans-18:1 to the microbial community [39]. 

The increase in t11, c15-18:2 and c15-18:1 in the abomasal digesta of BAO lambs is also surprising. These FA are both exclusive intermediates of C18:3n-3 BH pathways, but the BAO diet led to the lowest intake of 18:3n-3 and of 18:3n-3 BH, which should lead to a decreased proportion of t11, c15-18:2 and c15-18:1. Nevertheless, our results seem to be consistent with those reported by Panyakaew et al. [41], which showed that MCFA disturbed the BH of 18:3n-3 leading to an accumulation of t11, c15-18:2.

## 5. Conclusions

Feeding BAO reduced DM intake without affecting the fiber digestibility. The total FA apparent digestibility of the BAO diet was greater when compared with the CON and BUO diets due to the greater digestibility of 12:0. However, BAO increased the t10-18:1 in the abomasal content, even with low biohydrogenation intermediates, leading to possible stress for ruminal bacteria.

## Figures and Tables

**Table 1:** Ingredients, chemical composition and fatty acid profile of experimental diets.

Ingredients, g/kg DM	Diets ^1^		
	CON	BAO	BUO
Tifton hay	300.0	300.0	300.0
Ground corn	460.0	415.0	415.0
Soybean meal	210.0	215.0	215.0
Oil	0.0	40.0	40.0
Limestone	5.0	5.0	5.0
Mineral premix ^2^	25.0	25.0	25.0
Chemical composition, g/kg DM			
Dry matter	906.8	909.0	910.8
Organic matter	948.5	949.5	947.0
Crude protein	161.3	165.5	160.0
Neutral detergent fiber	404.8	396.3	394.6
Non-fiber carbohydrates	344.7	324.1	325.6
Metabolizable energy (MJ/kg)	26.6	29.1	27.1
Fatty acids, g/kg of DM			
8:0	0.00	1.65	0.00
10:0	0.00	1.96	0.00
12:0	0.00	18.85	0.01
14:0	0.00	6.22	0.03
16:0	0.66	3.59	7.59
18:0	0.08	1.32	0.68
c9-18:1	0.88	5.23	30.79
18:2 n-6	1.75	2.19	2.26
18:3 n-3	0.17	0.12	0.54
20:0	0.02	0.34	0.05
22:0	0.01	0.01	0.03
24:0	0.01	0.02	0.03
Others ^3^	0.02	0.02	0.79
Saturated fatty acids	0.79	33.96	8.48
Monounsaturated fatty acids	0.91	5.25	31.53
Polyunsaturated fatty acids	1.86	2.28	2.80
Total fatty acid, g/kg of DM	3.56	41.52	42.81

^1^ CON = diet without added oil; BAO = 40 g/kg DM of babassu oil; BUO = 40 g/kg DM of buriti oil; ^2^ Composition: Ca 22%, P 5.5%, Mg 3.5%, S 2.2%, Cl 10.5%, Na 7.0%, Mn 1500 mg/kg, Fe 500 mg/kg, Zn 1550 mg/kg, Cu 440 mg/kg, Co 50 mg/kg, I 40 mg/kg, Se 20 mg/kg; ^3^ Sum of C15:0, C16:1, C17:0, C18:1 cis 11, C21:1 and C23:0.

**Table 2:** Fatty acid profile (g/kg total FA) of babassu oil and buriti oil.

Fatty Acid	Babassu	Buriti
8:0	42.8	2.8
10:0	51.0	-
12:0	490	-
14:0	162	0.81
16:0	80.0	178
18:0	32.5	15.2
*c*9-18:1	116	757
*c*11-18:1	-	11.4
18:2n-6	16.6	16.5
18:3n-3	3.2	10.9
Others ^1^	8.8	10.0
Total FA (g/kg oil)	979	976

^1^ Sum of 16:1; 17:0; 20:0; 22:0; 20:1; 23:0; and 24:0.

**Table 3:** Ruminal pH, intake and nutrient digestibility of sheep fed diets containing babassu or buriti oil.

Item ^1^	Diets ^2^			SEM ^3^	*p*-Value
	CON	BAO	BUO		
Dry matter					
Intake (g/day)					
Dry matter	1068 ^a^	804 ^b^	977 ^a^	40.1	0.014
Crude protein	201 ^a^	154 ^b^	181 ^a^	7.3	0.024
NDF	382 ^a^	280 ^b^	337 ^a^	14.7	0.008
Total CHO	785 ^a^	563 ^b^	690 ^a^	30.7	0.005
NF-CHO	402 ^a^	284 ^b^	353 ^a^	16.1	0.004
uuME (MJ/d)	28.4	23.7	26.2	0.95	0.118
Apparent digestibility (g/kg)					
Dry matter	727	753	752	11.0	0.507
Crude protein	725	777	754	13.8	0.216
NDF	578	598	630	17.1	0.490
Total CHO	750	759	777	10.6	0.590
NF-CHO	905	924	927	7.8	0.464
Rumen pH	5.81 ^b^	5.50 ^c^	6.02 ^a^	0.081	<0.001

Means within a row with different superscripts are significantly different (*p* < 0.05); ^1^ NDF: Neutral detergent fiber; CHO: carbohydrate; NF-CHO: non fibrous carbohydrate; ME: metabolizable energy; ^2^ CON = diet without added oil; BAO = 40 g/kg DM of babassu oil; BUO = 40 g/kg DM of buriti oil; ^3^ SEM = Standard error of the mean.

**Table 4:** Intake and apparent digestibility of fatty acids of sheep fed diets containing babassu or buriti oil.

Item	Diets ^1^			*p*-Value
	CON	BAO	BUO	
Intake (g/day)				
12:0	-	16.5 ± 0.85	-	-
16:0	0.71 ± 0.073 ^c^	3.14 ± 0.468 ^b^	7.66 ± 0.468 ^a^	<0.001
18:0	0.08 ± 0.067 ^b^	1.14 ± 0.053 ^a^	0.67 ± 0.062 ^c^	<0.001
*c*9-18:1	0.95 ± 0.261 ^c^	4.57 ± 0.197 ^b^	31.08 ± 2.603 ^a^	<0.001
18:2 n-6	1.87 ±0.205	1.91 ± 0.106	2.27 ± 0.192	0.231
18:3 n-3	0.18 ± 0.050 ^b^	0.10 ± 0.005 ^b^	0.54 ± 0.038 ^a^	<0.001
C18 FA	3.13 ±0.471 ^c^	7.77 ± 0.356 ^b^	35.06 ± 2.937 ^a^	<0.001
Total FA	3.30 ±1.76 ^c^	27.10 ± 1.45 ^b^	42.40 ±3.62 ^a^	<0.001
Apparent digestibility (g/kg)				
12:0	-	994 ± 0.6	-	-
16:0	−67.8± 180.7 ^b^	690 ± 51.9 ^a^	763 ± 51.9 ^a^	0.030
18:0	−9484 ±2284 ^b^	−1102 ±1970 ^a^	−12,116 ±1970 ^b^	0.005
*c*9-18:1	836 ± 47.6 ^c^	968 ± 3.0 ^b^	990 ± 3.00 ^a^	<0.001
18:2 n-6	905± 42.0	961± 7.04	942± 7.04	0.121
18:3 n-3	801 ± 39.4 ^b^	729 ± 29.8 ^b^	943 ± 5.71 ^a^	<0.001
SC18 FA ^2^	787± 99.2	682± 98.3	763 ± 65.4	0.725
Total FA	711 ± 23.9 ^b^	842 ± 41.5 ^a^	765 ± 72.3 ^ab^	0.047

Means within a row with different superscripts are significantly different (*p* < 0.05); ^1^ CON = diet without added oil; BAO = 40 g/kg DM of babassu oil; BUO = 40 g/kg DM of buriti oil. ^2^ Sum of C18:0; *c*9-18:1; *c*11-18:1; C18:2 n-6; and C18:3 n-3.

**Table 5:** Total fatty acid (FA) content (mg/g DM) and composition (g/kg total FA) of the abomasal content of sheep fed diets containing babassu or buriti oil.

Item	Diets ^1^			SEM	*p*-Value
	CON	BAO	BUO		
Total FA content	42.9 ^b^	49.0 ^b^	59.6 ^a^	2.54	0.023
FA composition					
10:0	-	1.51 ^a^	0.04 ^b^	0.176	0.003
12:0	6.10 ^b^	52.4 ^a^	3.24 ^b^	5.742	0.023
13:0	0.82 ^a^	0.56 ^b^	0.54 ^b^	0.050	<0.001
i-14:0	2.60 ^a^	1.53 ^b^	1.71 ^b^	0.131	<0.001
14:0	5.75 ^c^	86.4 ^a^	11.9 ^b^	8.516	<0.001
i-15:0	8.00 ^a^	3.27 ^b^	4.43 ^b^	0.508	0.023
a-15:0	14.9 ^a^	7.7 ^b^	7.2 ^b^	0.857	0.041
15:0	8.00 ^a^	3.20 ^b^	4.44 ^b^	0.655	0.036
i-16:0	2.68 ^a^	1.23 ^b^	2.13 ^ab^	0.230	0.003
16:0	174 ^ab^	202 ^a^	171 ^b^	3.811	0.041
i-17:0	3.77 ^a^	2.35 ^b^	1.85 ^b^	0.233	0.042
a-17:0 + *c*9-16:1	4.50 ^a^	3.20 ^b^	2.61 ^b^	0.266	0.028
17:0	5.88 ^a^	4.54 ^ab^	3.82 ^b^	0.377	0.037
i-18:0	0.68 ^a^	0.17 ^b^	0.74 ^a^	0.105	0.008
18:0	626 ± 17.429 ^b^	420 ± 51.863 ^c^	685 ± 17.405 ^a^		0.001
*t*6-/*t*7-/*t*8-18:1	2.60	4.02	1.98	0.244	0.071
*t*9-18:1	1.64	1.91	1.10	0.175	0.675
*t*10-18:1	3.73 ± 0.432 ^b^	71.0± 31.036 ^a^	2.40 ± 0.435 ^c^		0.002
*t*11-18:1	18.1 ^a^	6.01 ^b^	6.20 ^b^	2.408	0.027
*t*12-18:1	2.81 ^a^	1.81 ^b^	1.82 ^b^	0.189	0.003
*c*9-18:1	36.8	36.8	45.7	4.431	0.265
*t*15-18:1	2.40 ^a^	1.82 ^b^	1.55 ^b^	0.167	0.017
*c*11-18:1	3.04 ^b^	5.44 ^a^	2.67 ^b^	5.104	<0.001
*c*12-18:1	0.11 ^b^	0.70 ^a^	0.51 ^a^	0.085	0.023
*c*13-18:1	0.28 ^a^	0.2 ^ab^	0.1 ^b^	0.029	0.051
*t*16-/*c*14-18:1	3.34 ^a^	1.47 ^c^	2.22 ^b^	0.183	<0.001
*c*15-18:1	0.35 ^b^	1.18 ^a^	0.22 ^b^	0.137	<0.001
*t*11 *c*15-/*t*10 *c*15-18:2	0.13 ± 0.016	0.13 ± 0.662	0.19 ± 0.660		0.235
18:2n- 6	2.31 ± 2.420 ^ab^	3.60 ± 6.836 ^a^	1.66 ± 2.414 ^b^		0.028
20:0	7.94 ^a^	5.04 ^c^	6.64 ^b^	0.296	0.025
20:1	0.33 ^b^	0.72 ^a^	0.37 ^b^	0.062	0.024
18:3n-3	5.48 ^a^	6.44 ^a^	3.06 ^b^	0.418	0.047
22:0	4.04 ^a^	2.65 ^b^	2.17 ^c^	0.187	<0.001
23:0	1.57 ^a^	1.00 ^b^	0.84 ^c^	0.074	<0.001
24:0	5.77 ^a^	3.88 ^b^	3.00 ^c^	0.262	0.026
26:0	2.67 a	1.71 ^b^	1.42 ^c^	0.129	0.030
oxo-18:0	2.03 ± 0.594 ^c^	11.3 ± 4.956 ^a^	5.56 ± 0.590 ^b^		0.002
FA Sums					
SFA ^2^	854 ^ab^	785 ^b^	886 ^a^	16.25	0.001
BCFA ^3^	37.2 ^a^	19.5 ^b^	20.8 ^b^	1.893	0.028
*cis*–MUFA ^4^	41.6	44.4	49.3	4.471	0.170
*trans*–MUFA	34.8 ± 3.562 ^b^	93.1 ± 36.546 ^a^	16.6 ± 3.560 ^b^		0.003
PUFA ^5^	29.0 ^b^	43.9 ^a^	20.0 ^b^	3.537	0.041
BI ^6^	38.7 ± 3.878 ^b^	108 ± 41.36 ^a^	23.2 ± 3.877 ^c^		0.013
FA Ratios					
*t*10/*t*11	0.21 ± 0.165 ^c^	5.24 ± 1.234 ^a^	0.71 ± 0.165 ^b^		0.002

Means within a row with different superscripts are significantly different (*p* < 0.05); ^1^ CON = diet without added oil; BAO = 40 g/kg DM of babassu oil; BUO = 40 g/kg DM of buriti oil; SEM = Standard error of the mean; ^2^ SFA = saturated fatty acids; ^3^ BCFA = Branched chain fatty acids; ^4^ MUFA = Monounsaturated fatty acids; ^5^ PUFA = Polyunsaturated fatty acids; ^6^ BI = Biohydrogenation intermediates (Sum of t6-/t7-/t8-, t9-, t10-, t11-, t12-, t15-, c12-, c13-, t16-/c14-, c15-18:1 isomers, t11,c15-/t10,c15-18:2 and oxo-18:0).

**Table 6:** Rumen biohydrogenation indicator estimates for lambs fed diets containing babassu or buriti oil.

Biohydrogenation Extent (%)	Diets ^1^			SEM ^2^	*p*-Value
	CON	BAO	BUO		
*c*9-18:1	83.6 ^b^	93.3 ^a^	89.6 ^a^	1.37	0.024
18:2n-6	95.5 ^a^	78.0 ^b^	70.1 ^b^	2.92	0.001
18:3n-3	81.8 ± 2.14 ^a^	23.8 ± 7.45 ^c^	74.8 ± 2.14 ^b^		<0.001
Completeness	93.9 ^a^	77.3 ^b^	97.3 ^a^	3.24	0.006

Means within a row with different superscripts are significantly different (*p* < 0.05); ^1^ CON = diet without added oil; BAO = 40 g/kg DM of babassu oil; BUO = 40 g/kg DM of buriti oil; ^2^ SEM = Standard error of the mean.

## Data Availability

Not applicable.
